# *QuickStats:* Age-Adjusted Suicide Rates,*^,†^ by State — National Vital Statistics System, United States, 2018

**DOI:** 10.15585/mmwr.mm6917a4

**Published:** 2020-05-01

**Figure:**
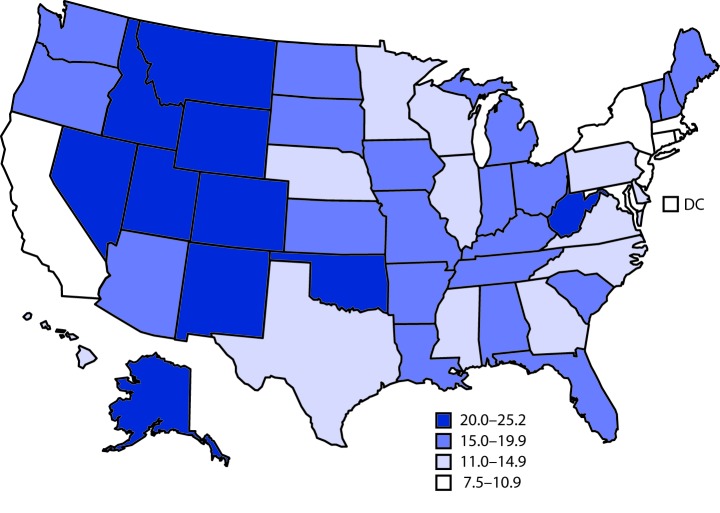
In 2018, the U.S. suicide rate was 14.2 per 100,000 standard population, with rates varying by state. The five states with the highest age-adjusted suicide rates were Wyoming (25.2), New Mexico (25.0), Montana (24.9), Alaska (24.6), and Idaho (23.9). The five jurisdictions with the lowest suicide rates were the District of Columbia (7.5), New Jersey (8.3), New York (8.3), Rhode Island (9.5), and Massachusetts (9.9).

**Source:** National Vital Statistics System. Underlying cause of death data, 1999–2018. https://wonder.cdc.gov/ucd-icd10.html.

For more information on this topic, CDC recommends the following link: https://www.cdc.gov/violenceprevention/suicide/index.html.

